# Loss of Acetylcholine Signaling Reduces Cell Clearance Deficiencies in *Caenorhabditis elegans*

**DOI:** 10.1371/journal.pone.0149274

**Published:** 2016-02-12

**Authors:** Sérgio M. Pinto, Johann Almendinger, Juan Cabello, Michael O. Hengartner

**Affiliations:** 1 Institute of Molecular Life Sciences, University of Zurich, Zurich, Switzerland; 2 Graduate Program in Areas of Basic and Applied Biology, University of Porto, Porto, Portugal; 3 Center for Biomedical Research of La Rioja (CIBIR), Logroño, La Rioja, Spain; Brown University/Harvard, UNITED STATES

## Abstract

The ability to eliminate undesired cells by apoptosis is a key mechanism to maintain organismal health and homeostasis. Failure to clear apoptotic cells efficiently can cause autoimmune diseases in mammals. Genetic studies in *Caenorhabditis elegans* have greatly helped to decipher the regulation of apoptotic cell clearance. In this study, we show that the loss of levamisole-sensitive acetylcholine receptor, but not of a typical neuronal acetylcholine receptor causes a reduction in the number of persistent cell corpses in worms suffering from an engulfment deficiency. This reduction is not caused by impaired or delayed cell death but rather by a partial restoration of the cell clearance capacity. Mutants in acetylcholine turn-over elicit a similar phenotype, implying that acetylcholine signaling is the process responsible for these observations. Surprisingly, tissue specific RNAi suggests that UNC-38, a major component of the levamisole-sensitive receptor, functions in the dying germ cell to influence engulfment efficiency. Animals with loss of acetylcholine receptor exhibit a higher fraction of cell corpses positive for the “eat-me” signal phosphatidylserine. Our results suggest that modulation by ion channels of ion flow across plasma membrane in dying cells can influence the dynamics of phosphatidylserine exposure and thus clearance efficiency.

## Introduction

Efficient clearance of dead cells is key for proper organismal health and homeostasis. Dying cells that are not properly removed can release harmful cellular contents into the surrounding tissue, potentially resulting in inflammation and autoimmune disease [1, 2].

The nematode *Caenorhabditis elegans* has been used with great success as a model to study the molecular basis of apoptosis and cell clearance. Apoptotic cells in *C*. *elegans* are rapidly engulfed and digested. As there are no professional phagocytes in *C*. *elegans*, this function is performed by adjacent cells [3]. For example, apoptotic germ cells are engulfed by the surrounding gonadal sheath cells [4]. As is the case in mammals, dying cells in *C*. *elegans* expose the phospholipid phosphatidylserine (PS) as an “eat me” signal [5, 6]. PS is normally restricted to the inner leaflet of the plasma membrane. Upon apoptotic stimulus, PS is translocated to the outer leaflet, signaling the presence of the doomed cell [7, 8]. Recently CED-8, the *C*. *elegans* homolog of the mouse lipid scramblase Xk-related protein 8, has been shown to play a critical role in PS exposure upon apoptotic stimuli; inability to efficiently expose PS results in delayed or impaired engulfment [9, 10]. PS is thought to be recognized by the engulfing cell through the transmembrane receptor CED-1 (LRP1/MEGF10), which binds PS via the bridging molecule TTR-52 [11]. The adapter protein CED-6 (GULP) relays the CED-1 signal downstream to regulate phagosome maturation through DYN-1 (Dynamin) and corpse engulfment through the small GTPase CED-10 (Rac1) [12–14]. Two further pathways act partially redundantly with the CED-1 pathway to promote corpse clearance in *C*. *elegans* [13, 15]. In the first pathway, UNC-73 (Trio), a guanosine exchange factor (GEF), activates the small GTPase MIG-2 (RhoG), which in turn regulates corpse removal by modulating activation of the bipartite GEF formed by CED-5 (Dock180) and CED-12 (Elmo) [16, 17]. A third protein, CED-2 (CrkII), further stabilizes the CED-5/CED-12 complex, allowing downstream activation of CED-10 [18–20]. Another pathway involves ABL-1 (Abl kinase), which negatively regulates engulfment by inhibiting ABI-1 (Abi). ABI-1 promotes cell clearance either by regulating CED-10 activity or through an independent pathway [15]. Active CED-10 leads to cytoskeleton reorganization, corpse internalization and ultimately degradation of the engulfed corpse [2, 13].

Variations in intracellular ion concentration, in particular Ca^2+^, have been shown to influence the efficiency of apoptotic cell clearance [21, 22]. Ligand-gated ion channels are an important family of protein complexes involved in neurotransmission that can influence and be influenced by local ion concentrations [23]. Studied for more than a century, the nicotinic acetylcholine receptor (nAChR) is an example of such a complex. In vertebrates, nAChRs assemble mostly in heteropentameric channels, formed in the central nervous system and in the neuromuscular junction by two alpha and three non-alpha subunits, or less frequently in homopentameric structures in the CNS composed of only alpha subunits (α7) [24, 25]. Individual subunits contain four membrane-spanning domains with the transmembrane domain 2 lining in the pore channel [24].

*C*. *elegans* has an extensive nAChR family, composed of at least 29 subunit-encoding genes [26, 27]. The levamisole-sensitive receptor (levamisole receptor), is the best-studied nAChR in *C*. *elegans*. It is expressed in the body-wall muscle, where it works as the main excitatory receptor at the neuromuscular junction. The anthelmintic levamisole acts as an agonist of the levamisole receptor: exposure to levamisole results in tetanus (hypercontraction and paralysis of the striated body-wall muscles), and ultimately death of the organism [28]. Mutants in subunits of the levamisole receptor were among the first nAChR subunits to be identified, mainly because they confer partial or complete resistance to levamisole [28]. These studies identified two non-alpha subunits (LEV-1 and UNC-29) and three α subunits (UNC-38, UNC-63 and LEV-8).

Given the existence of a high number of *C*. *elegans* AChR subunits, different receptor compositions can occur. These composition variations seem to be important for channel specificity, place of expression, and function [26]. Thus, while UNC-38 and UNC-63 are for example present in both the levamisole and the ACR-2R (neuronal) receptor, LEV-1, LEV-8, and UNC-29 are specific for the levamisole receptor, whereas other subunits such as ACR-2, ACR-3, ACR-12 are only found in neuronal receptors [29]. ACR-8 a levamisole-independent subunit can be found in both tissues [30]. In vertebrates, all the channels are permeable to the cations Na^+^, K^+^ and Ca^2+^ with the permeability to the three ions varying among the different receptors. Whereas Ca^2+^ flux in the muscular receptor is low, it can be quite high in neuronal receptors, depending on subunit composition [23, 31]. The *C*. *elegans* levamisole receptor has been shown to be permeable to Ca^2+^ [32].

In vertebrates, nAChRs are also present in many non-neuronal and non-muscular tissues such as bronchial epithelial cells and macrophages [33, 34], suggesting that nAChRs may have functions beyond neuronal and neuro-muscular signaling.

Here we report our observation that in worms deficient in engulfment, loss of acetylcholine (ACh) signaling results in a reduction in the number of cell corpses. We show that this reduction is not due to lower apoptotic activity or delayed timing of cell death but rather due to a partial restoration of engulfment efficiency. Indeed, in worms lacking proper ACh signaling, a higher percentage of cell corpses are PS-positive. Taken together, our results suggest that modulation by ion channels of the ionic flow across the plasma membrane of apoptotic cells can affect the dynamics of PS exposure and thus clearance efficiency.

## Materials and Methods

### Strains and mutations

*C*. *elegans* strains where maintained using standard conditions [35]. The Bristol N2 strain was used as wild type. The following alleles were used: LGI: *ace-2(ok2545)*, *unc-38(x20)*, *unc-63(ok1075)*, *ced-12(k149)*, *ced-12(oz167)*, *rrf-1(pk1417)*, *unc-29 (e1072)*, *ced-1(e1735)*, *ced-1(n1995)*, *unc-54(e1092)*. LGIII: *ced-6(n1813)*, *ced-6(tm1826)*, *ced-7(n1892)*, *ced-7(n1996)*, *mut-7(pk204)*. LGIV: *ced-2(e1752)*, *ced-2(n1994)*, *ced-10(n1993)*, *ced-10(n3246)*, *cha-1(ok2253)*, *unc-17(e245)*, *ced-5(n1812); ced-5(tm1949)*, *cho-1(ok1069)*. LGV: *unc-76(e911)*. LGX: *ced-8(n1891)*, *ace-1(ok663)*.

The following integrated array containing [*unc-76(+)*] was used: *smIs76(P*_*hsp-16*.*41*_::*sAnxV*::*gfp]* [36].

Description of all alleles can be found in WormBase (http://www.wormbase.org/).

### Cell corpse analysis

Embryonic apoptotic cell corpses: adult worms were dissected and the embryos were mounted on a 3% agar pad in a drop of M9 buffer. Developing embryos were recorded at 20°C for 15 hours with ∼35 different focal planes (0.69 μm separation) every 60 seconds (4D microscopy), using a Leica DM6000B equipped with Nomarski optics. Apoptotic cell corpses were scored at the following embryonic developmental stages: “ball of cell” (200 minutes post-fertilization), “tram-track” (280 minutes), “u-view” (360 minutes), “bean” (385 minutes), 1.5-fold (420 minutes), 2-fold (450 minutes), 3-fold (510 minutes), 4-fold (600 minutes) and hatching (800 minutes).

Larval L1 head apoptotic cell corpses: plates containing adult worms and embryos were washed thoroughly with M9 buffer so that only embryos remained on the plates. 50 minutes later freshly hatched L1 larvae were mounted on a 3% agar pad, anesthetized (2.5 mM levamisole in M9 buffer), and immediately scored for apoptotic cell corpses in the head region using a microscope equipped with Nomarski optics.

Germ-cell corpses: 12 hours post-L4/adult molt, animals were mounted on a 3% agar pad and anesthetized in a droplet of 5 mM levamisole in M9, and apoptotic germ cell corpses were scored using a microscope equipped with Nomarski optics.

### Cell survival in the pharynx

L3 and L4 stage animals were mounted on a 3% agar pad, anesthetized in a droplet of 5 mM levamisole in M9, and scored in the procorpus and the metacorpus for extra cell nuclei as described [37].

### Lineaging and cell corpse clearance time quantification

Embryos were prepared and mounted as previously described [3]. Developing embryos were recorded at 25°C for 10 hours with 25 different focal planes (1 μm separation) every 60 seconds (4D microscopy), using a Zeiss Axioplan microscope equipped with Nomarski optics. Embryo lineages were determined using the software SIMI Biocell (SIMI GmbH, Germany) as described [38]. Cell corpse clearance time is defined as follows: t_death_ = onset of cell death (refractive corpse starts to be seen); t_clea r_ = refractive corpse is no longer visible (tiny granule); cell corpse clearance time = t_clear_−t_death_.

### RNA interference (RNAi)

RNAi was performed as previously described [39]. NGM agarose plates containing 2mM IPTG (Isopropyl β-D-1-thiogalactopyranoside) were allowed to dry overnight at room temperature (∼12 hours) after being seeded with 250 μl of appropriate bacterial strains. Approximately 50 staged L1 larvae of the desired genotype were transferred to the plate and allowed to grow at 20°C.

### PS exposure test

Worms carrying the *smIs76* reporter were incubated at 33°C for 45 minutes, and then allowed to recover for 2 hours at 20°C. “Bean” and 4-fold stage embryos were recorded as described above. Both DIC and fluorescent images were captured in succession. The percentage of AnxV::GFP positive corpses is the fraction of corpses visible by DIC that also showed AnxV::GFP rings.

## Results

### Loss of *C*. *elegans* levamisole acetylcholine receptor subunits causes reduction of persistent cell corpses in engulfment-defective backgrounds

**Fig 1:**
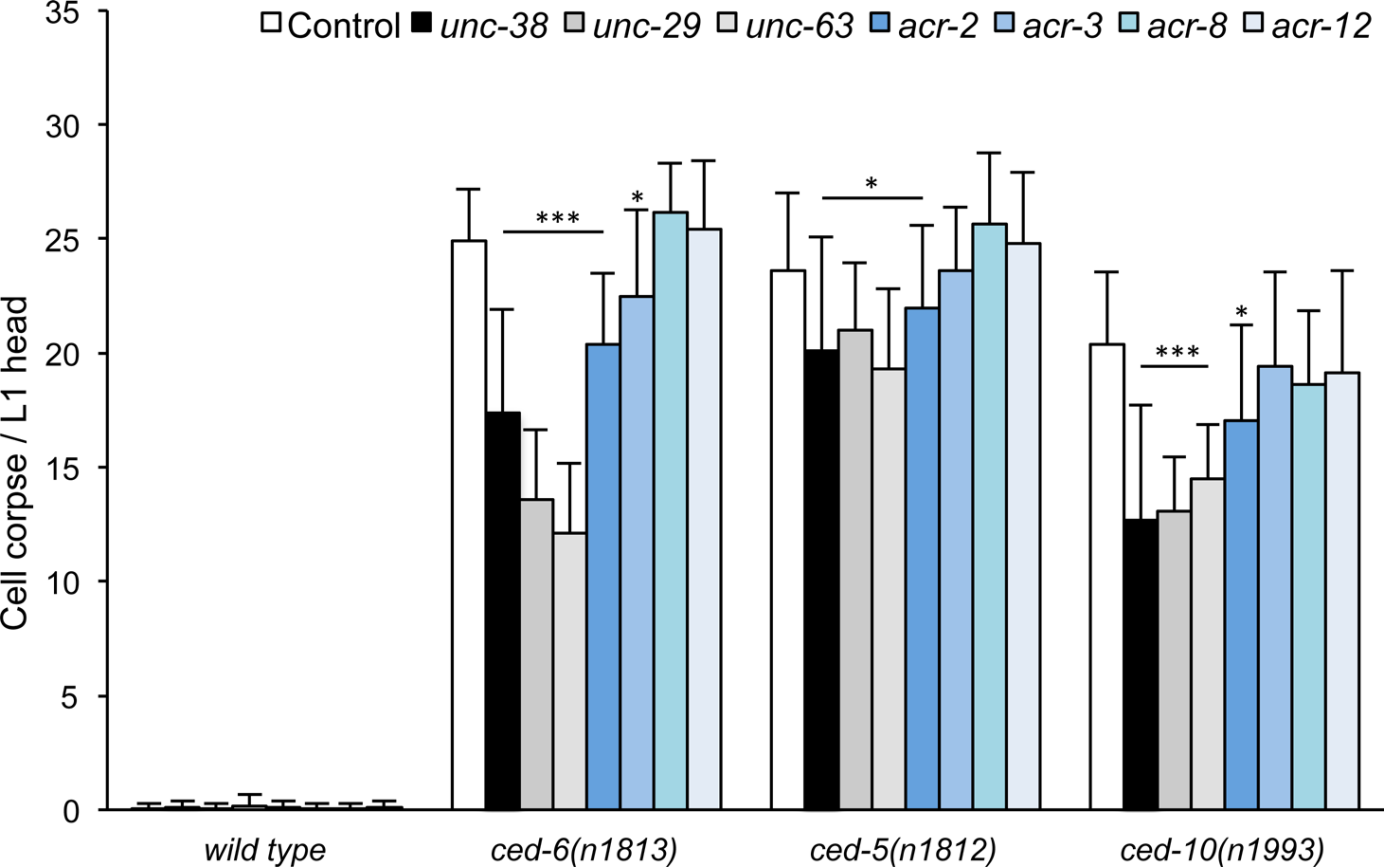
Loss of subunits of the levamisole receptor but not of other nAChRs reduces cell corpse numbers. Cell corpses were scored in the head region of freshly hatched L1 larvae of the indicated genotypes. *ced-x* stands for the genotypes on the x-axis. Data shown are average ± standard deviation, n = 20.*p<0.05; **p<0.01; ***p<0.005, determined by *t*-test.

Mutants in *C*. *elegans* AChR subunits on their own do not show persistent cell corpses (Fig 1). To determine if AchR signaling can influence corpse cell removal in a sensitized background, we performed a series of double mutant analyses: we crossed mutations in genes coding for subunits of the muscular (levamisole receptor) and various neuronal AChRs into three engulfment deficient backgrounds: *ced-5*, *ced-6 and ced-10*, representing the two classical engulfment pathways and the GTPase upon which they converge [13] and scored the number of persistent cell corpses in the L1 head. Mutations in the levamisole receptor subunits genes, *unc-38*, *unc-29* and *unc-63* resulted in an 11% to 51% decrease of the number of persistent cell corpses in all three engulfment backgrounds (Fig 1). By contrast mutation of various neuronal AChR subunits showed little to no effect.

### *unc-38* mutants show reduced apoptotic cell corpse number

**Fig 2:**
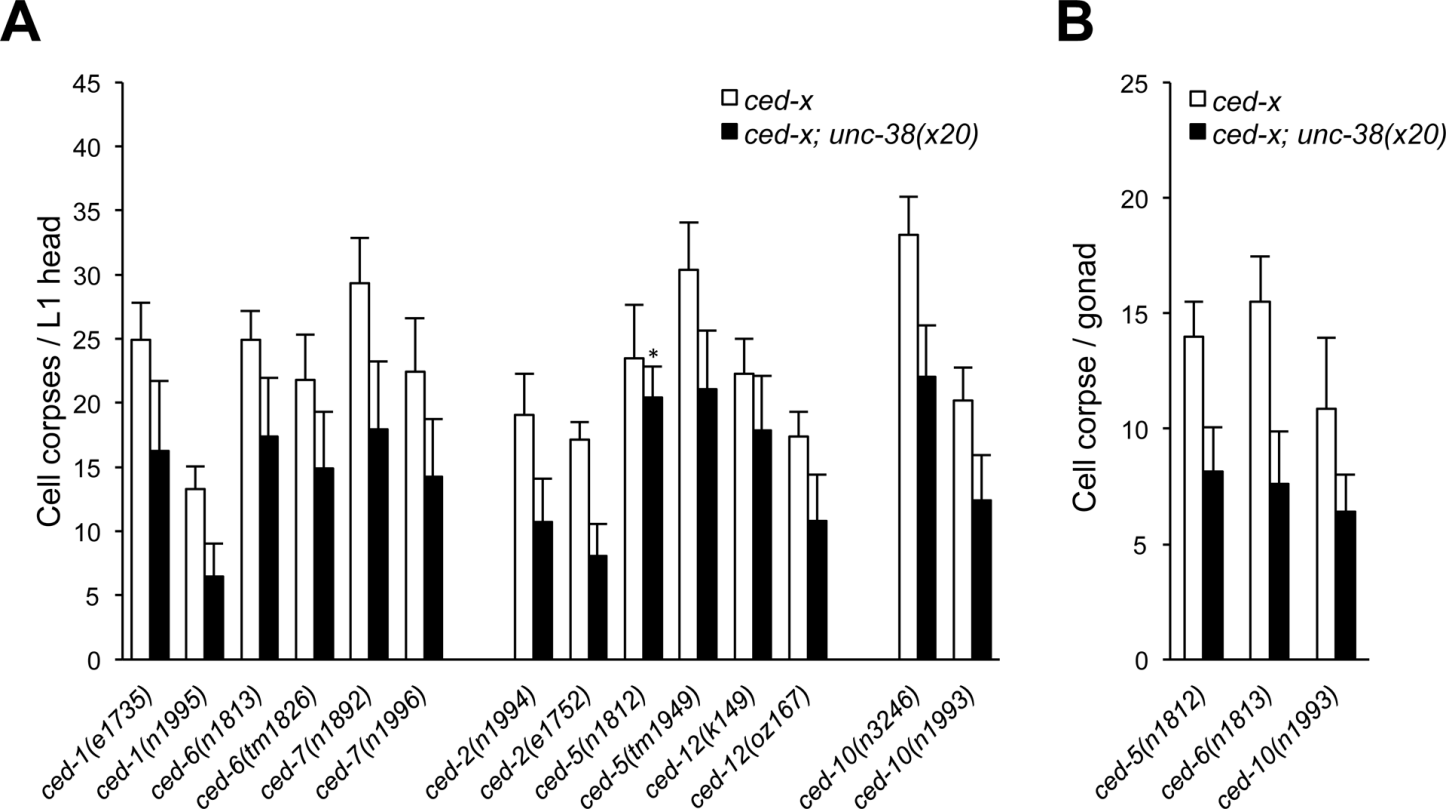
Loss of *unc-38* reduces cell corpse numbers in soma and germ lines of engulfment mutants. (A) Cell corpses were scored in the head region of freshly hatched L1 larvae of the indicated genotype. (B) Germ cell corpses were scored in adult animals of the indicated genotypes 12 hours post-L4/adult molt. *ced-x* stands for the genotypes on the x-axis. Data shown are average ± standard deviation, n = 20. All tests had a significance of *p*<0.005 with the exception of the case noted, *p<0.05, determined by *t*-test.

Since the strongest effects were observed with subunits from the levamisole receptor, we next tested the effect of loss of the major alpha-subunit of the levamisole receptor UNC-38 on cell corpse persistence in all of the major engulfment mutants from the two partially redundant pathways that mainly control corpse engulfment in *C*. *elegans*. Consistent with our preliminary observations, *unc-38* mutants showed a reduction in the number of persistent cell corpses in all engulfment-defective backgrounds tested (Fig 2A). This decrease ranged between a minimum of 13% in *ced-5(n1812)* to a maximum of 53% in the *ced-2(e1735)* background. We further analyzed the effect of loss of *unc-38* in the adult germline of *ced-5*, *ced-6* and *ced-10* mutants. As was the case in somatic tissues, loss of *unc-38* resulted in a reduction in persistent germ cell corpses (41% to 51%; Fig 2B). Loss of another subunit of the levamisole receptor, the non-alpha subunit UNC-29, similarly reduced in the number of corpses both in somatic tissues (12% to 64%) and the adult germline (23% to 41%) (S1 Fig).

**Fig 3:**
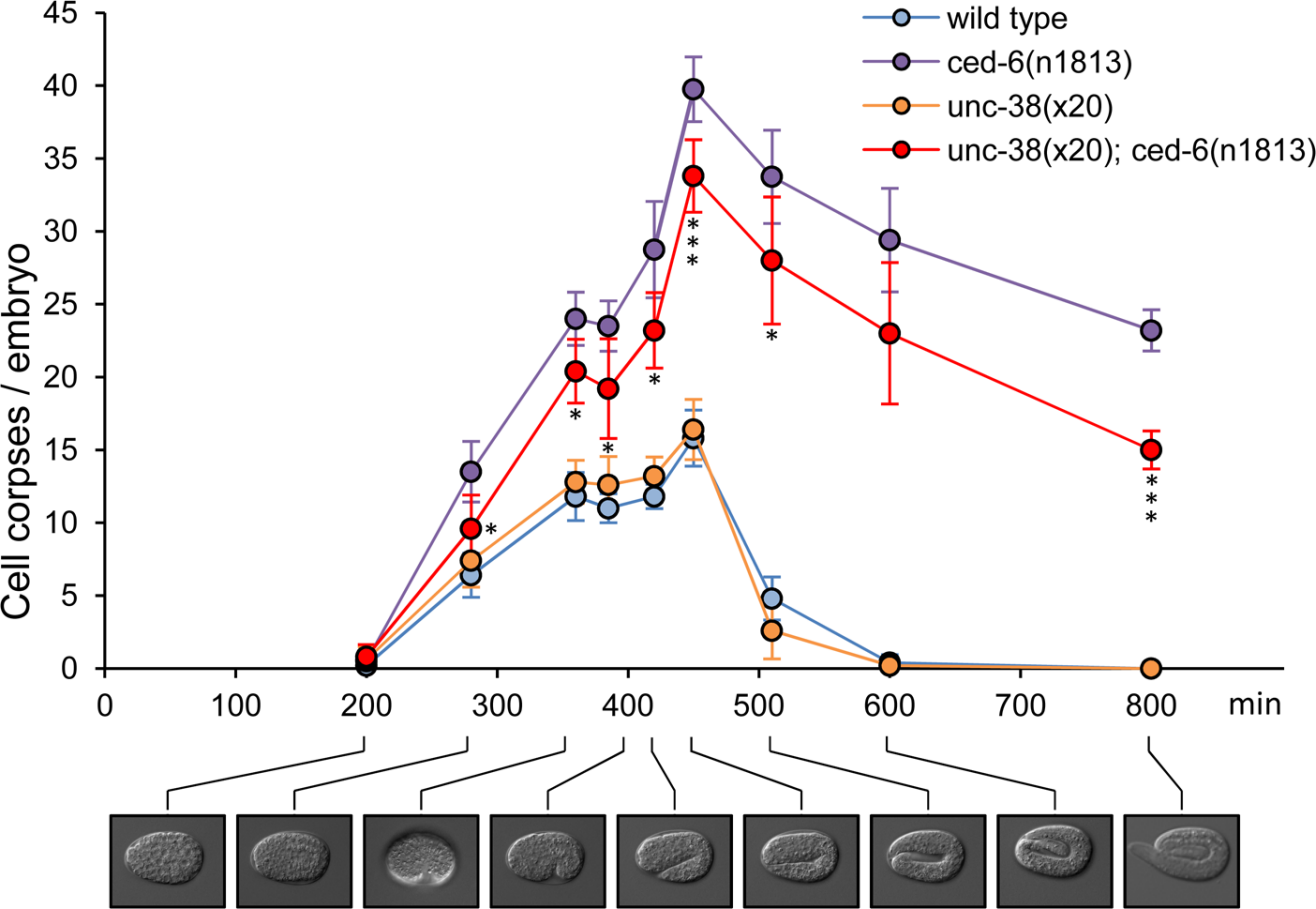
Loss of *unc-38* reduces cell corpse numbers in all embryonic developmental stages of engulfment mutants. 4D microscopy was performed on developing embryos of the indicated genotypes. The number of cell corpses was scored at discrete time points during embryonic development. Data shown are average ± standard deviation, n = 10. *p<0.05; **p<0.01; ***p<0.005, determined by *t*-test, between *ced-6* and *unc-38; ced-6*, no statistical significance was observed between wild type and *unc-38* mutants.

To better understand the cause of the reduction in persistent cell corpses, we analyzed the kinetics of cell corpse clearance during the embryonic development of *C*. *elegans*. In wild-type animals, apoptotic cells are rapidly removed from the tissue through engulfment by neighboring cells. In engulfment-defective worms such as *ced-6*, this process becomes inefficient, leading to a progressive accumulation of cell corpses, especially in late embryonic stages. Cell corpse numbers are slightly reduced when an *unc-38* mutation is introduced in *ced-6* mutant animals, reaching statistical significance at most but not all developmental stages tested (Fig 3). By contrast, *unc-38* had no significant effect on corpse numbers in a wild-type background in this assay (Fig 3).

### Kinetics and extent of apoptosis are not affected by loss of *unc-38* or *unc-29* function

**Fig 4:**
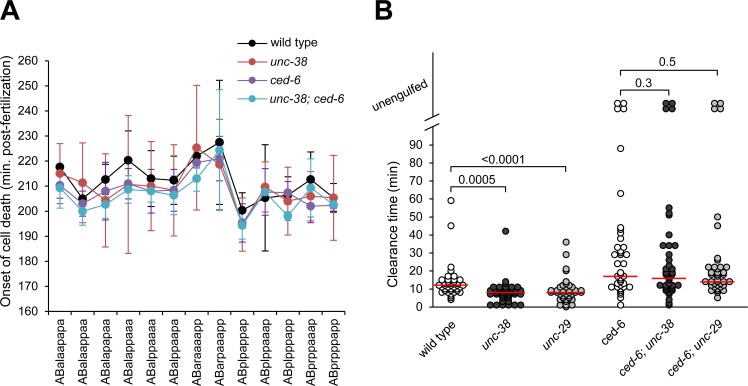
Apoptotic timing in *unc-38* and duration of cell corpse clearance in *unc-38* or *unc-29* mutants. (A) The time of onset of the first 13 apoptotic cell deaths of the AB lineage were followed by 4D microscopy. Data shown are average ± standard deviation. (B) Duration of cell corpses clearance was determined. Phenotype analysis was performed in three different animals of the indicated genotypes (n = 3 x 13 cells). Cells indicated as unengulfed floated off from the tissue into the egg-shell cavity or their lineage could no longer be followed due to the beginning of muscle contraction at the 1.5-fold stage (120 minutes post-onset of cell death). Each circle represents a single cell. Red lines mark the median. Alleles: *unc-38(x20)*, *unc-29(e1072)* and *ced-6(n1813)*. Statistical significance was assessed using the Wilcoxon signed-rank test.

A reduction in cell corpse number can be the result of a number of different conditions, including reduced apoptosis, delayed initiation of cell death or increased cell clearance [40]. To distinguish between these hypotheses, we quantified apoptotic activity in the anterior pharynx in the L3 larvae [37]. In wild-type animals, there are 49 cell nuclei in this region, but in apoptosis-defective animals, such as *ced-3* mutants, up to 12 additional nuclei can be observed [41]. Neither *unc-38* nor *unc-29* showed any extra nuclei in any of the animals tested (10 per genotype). Moreover, the onset of programmed cell death of the first 13 cells entering apoptosis from the AB embryonic lineage were normal (Fig 4A, S2 Fig), as assessed by 4D lineage [38]. Taken together, these results indicate that the observed reduction in persistent apoptotic cell corpses is not due to reduced levels of apoptosis or delayed activation of the cell death program.

### *unc-38* and *unc-29* mutants clear corpses faster

To determine if cell corpse reduction was due to an increase in the efficiency of clearance, we quantified the kinetics of death and clearance of the first 13 cell deaths from the AB lineage by 4D microscopy [38]. Wild-type animals took 13.4 ± 10.1 minutes (average ± standard deviation) to clear an apoptotic cell, while *unc-38* and *unc-29* took 8.1 ± 6.6 and 9.1 ± 7.3 minutes, respectively (p = 0.0005 for *unc-38* and p<0.0001 for *unc-29*, Wilcoxon signed-rank test, Fig 4B). This suggests that these mutants are able to clear corpses significantly faster than the wild type. Mutations in *ced-6* cause defects in corpses internalization that result in delayed cell clearance (23.3 ± 17.6 minutes, excluding uncleared cells). Surprisingly, unlike the situation in the single mutants, we found no significant statistical difference between *ced-6* and its double mutants with *unc-38* or *unc-29* (19.6 ± 13.9 and 17.3 ± 9.2 minutes respectively, Fig 4B). Taken together these results seem to point at a small increase in cell clearance efficiency in both *unc-38* and *unc-29* mutant animals. The weak effect observed by 4D lineage might in part be due to the fact that in this test cell corpse clearance is assessed very early in development, at a stage where the expression of the AChR genes is still weak [42].

### Mutations in genes controlling ACh turnover reduce the number of persistent cell corpses in engulfment-deficient backgrounds

**Fig 5:**
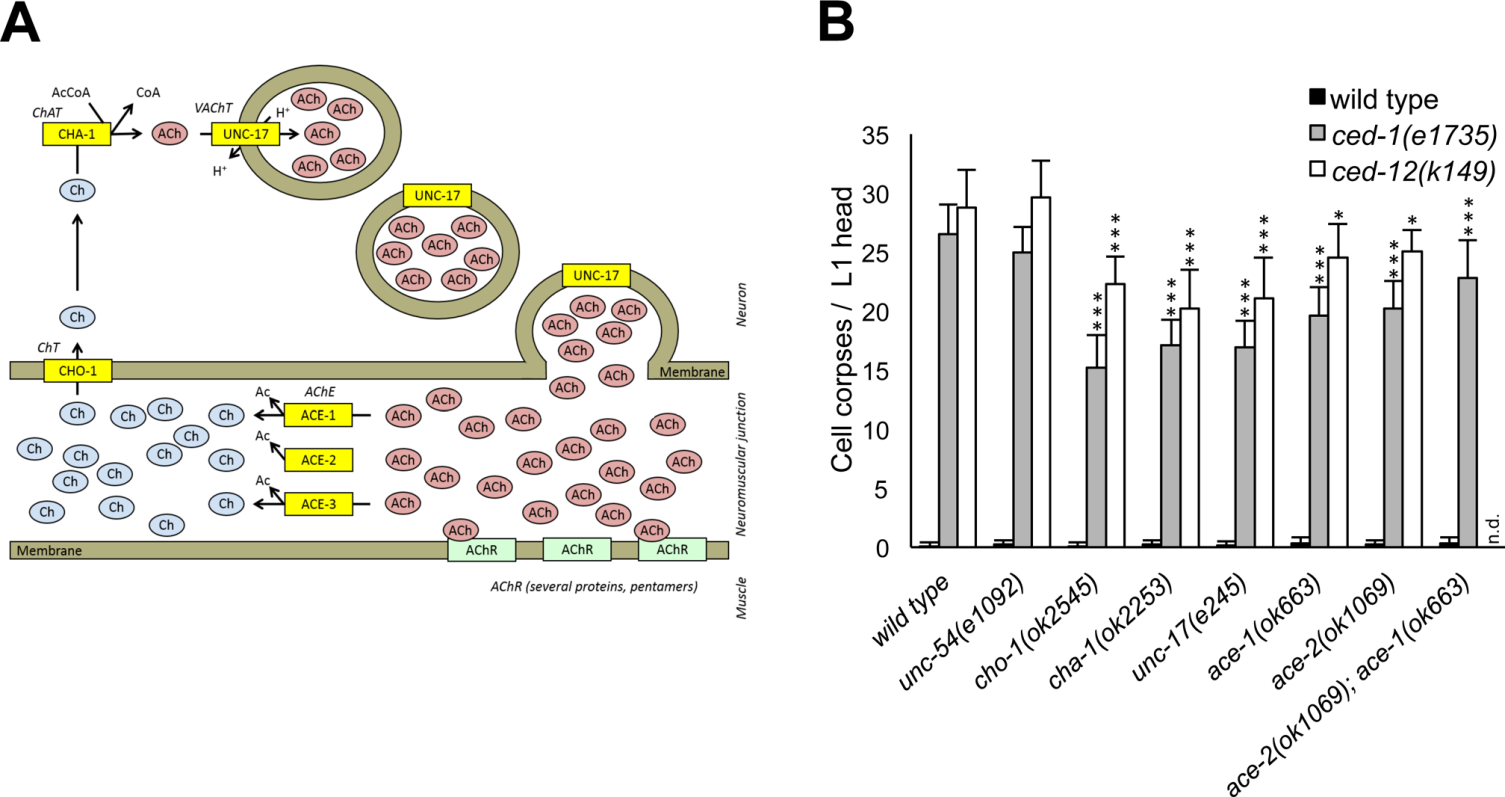
Loss of ACh synthesis and turnover results in a reduction of cell corpse numbers. (A) Cartoon of the neuromuscular junction of *C*. *elegans* highlighting the cholinergic enzymes and transporters responsible for the synthesis and turnover of acetylcholine. ChT–choline transporter, ChAT–choline acetyltransferase, VAChT–vesicular acetylcholine transporter, AChR–acetylcholine receptor, AChE–acetylcholinesterases, AcCoA–Acetyl coenzyme A, CoA–coenzyme A, Ac–acetyl group. (B) Cell corpses were scored in the head region of freshly hatched L1 larvae of the indicated genotype. Animals mutant in the UNC-54 muscle myosin were used as controls to rule out that the reduction of persistent cell corpses was due to the Unc phenotype. Data shown are average ± standard deviation, n = 20. *p<0.05; **p<0.01; ***p<0.005, determined by *t*-test.

Since loss of the major ACh receptor at the neuromuscular junction causes a reduction in the number of persistent cell corpses (Fig 1), we investigated if modulating ACh signaling through other means would result in a similar effect. In *C*. *elegans* the genes *cho-1*, *cha-1* and *unc-17* are responsible for the import of choline (Ch) into the cell, its acetylation and the loading of ACh into the synaptic vesicles, respectively (Fig 5A). Loss of any of these genes results in loss of proper ACh production and in reduced ACh signalling. On the other hand, three partially redundant acetylcholine esterases (AChE), *ace-1*, *ace-2* and *ace-3*, are responsible for the switch-off of ACh signaling through the hydrolysis of ACh back to Ch and acetate in the synaptic cleft [43]. Worms carrying mutations in all three genes simultaneously cannot survive, but double or single mutants are viable, even though double mutants grow slowly and have a severe impairment in locomotion, due to excessive ACh signalling [43]. Loss of *cho-1*, *cha-1* or *unc-17* led to a clear reduction in the number of apoptotic cell corpses, in both *ced-1* and *ced-12* engulfment deficient worms (Fig 5B). The same decrease was detected in the AChE single and double mutants. This result is surprising, as it suggests that both reduction as well as increase in ACh signaling can lead to increased engulfment activity. Since all the mutants in ACh signaling in this study show locomotion defects, we wished to rule out the possibility that the reduction in the number of persistent cell corpses was due to this fact. To this end, we quantified cell corpses in the muscle myosin mutant *unc-54*. No changes were observed in *unc-54* mutant worms in any of the tested backgrounds (Fig 5B), suggesting that loss of locomotion in not per se sufficient to alter engulfment activity.

### *unc-38* function is important in the dying cell

**Fig 6:**
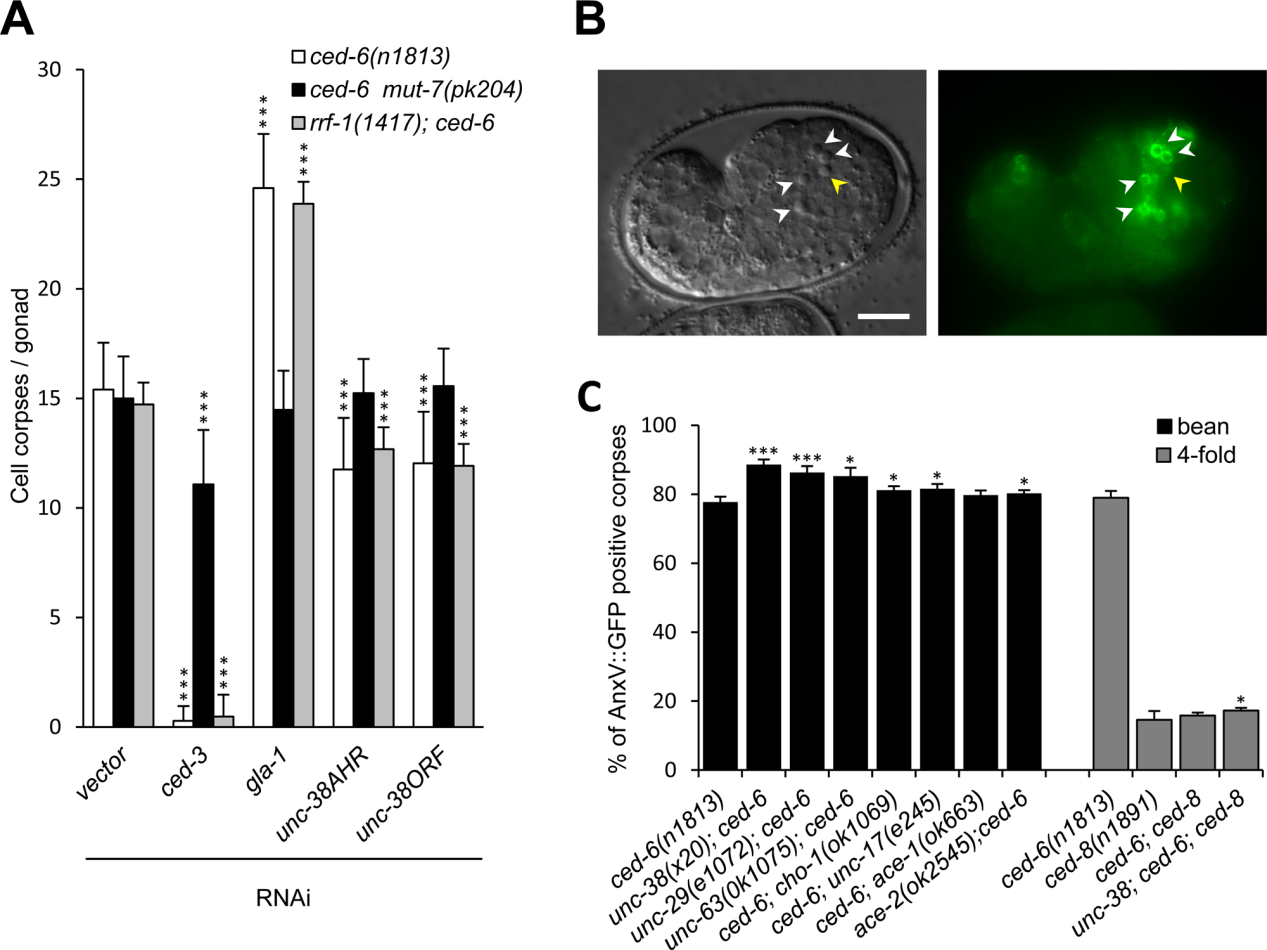
UNC-38 acts in germ cells (dying cells) to modulate engulfment efficiency. (A) Tissue-specific RNAi: cell corpses were scored in the germ line of animals of the indicated genotypes 12 hours post L4/adult molt. The positive controls *ced-3* and *gla-1* (reduced or increased corpse number) are known to act in dying germ cells [4, 13]. AHR–Ahringer RNAi library clone; ORF–Orfeome RNAi library clone. Error bars show standard deviation, * p<0.005, n = 20. (B) Left panel—DIC picture of an embryo at the “bean” stage; Right panel–AnxV::GFP reporter expression. White arrowheads show cell corpses positive for AnxV::GFP; yellow arrowhead shows a cell corpse negative for AnxV::GFP; scale bar: 10 μm. (C) Quantification of the fraction of germline corpses visible by DIC that are also positive for the AnxV::GFP reporter. Data shown are average ± standard deviation of 3 experiments (n≥10 worms, ≥200 corpses per experiment). *p<0.05; **p<0.01; ***p<0.005, determined by *t*-test.

In which cells must AChR act to influence cell corpse engulfment? To answer this question, we quantified germ cell death following *unc-38* RNAi in two different *C*. *elegans* mutants, *mut-7* and *rrf-1*, which are defective in RNAi in either the germline (the dying cell), or in the somatic tissue (the engulfing cell), respectively [44, 45]. Knock-down of *unc-38* specifically in germ cells (*rrf-1* background) still efficiently reduced the number of persistent germ cell corpses in *ced-6* mutants (Fig 6A). On the other hand, knock down of *unc-38* specifically in somatic tissue (*mut-7* background) was ineffective and gave a result similar to the negative control (vector alone) (Fig 6A). These results suggest that *unc-38* function is important in the dying cell rather than in the engulfing one, at least in the case of apoptotic germ cells.

### Defects in ACh signaling increase the fraction of PS-positive apoptotic cells

One of the major contributions of the dying cell towards its engulfment is the exposure of the PS “eat me signal” on the outer leaflet of its plasma membrane [5]. To test if the loss of ACh signaling genes influences PS exposure, we used the genetically encoded PS sensor Annexin V::GFP under the control of a heat-shock promoter to monitor PS during early embryonic development of *C*. *elegans* [36]. In our experimental setup, we looked at embryos at the “bean” stage 2 hours after heat shock (Fig 6B). In control worms of the *ced-6(n1813)* genotype, 77.7 ± 1.6% of the corpses identified by DIC microscopy were PS-positive. In the double mutant *ced-6(n1813); unc-38(x20)* a significantly higher (p = 0.0005) percentage of corpses (88.7 ± 1.5%), were PS-positive (Fig 6C). Increased numbers of PS-positive corpses could also be observed in the other two AChR subunits tested (*unc-29* and *unc-63*) and on three (*cho-1*, *unc-17* and *ace-2*) of the four ACh metabolic genes tested.

Thus, loss of *unc-38* and other ACh signaling genes result in a higher number of PS-positive corpses, suggesting that cell corpses can be more readily recognized by engulfing cells in *unc-38* mutants, possibly providing an explanation for the observed increase in engulfment efficiency in these mutants.

*C*. *elegans* CED-8 has been proposed to act as a lipid scramblase that promotes export of PS to the outer leaflet of the plasma membrane of apoptotic cells [9, 10]. We decided to investigate if the UNC-38 function in cell clearance acts through CED-8. Loss of *ced-8* results in marked impairment of PS exposure, both in a wild-type and an engulfment-defective background (Fig 6C and [10]).

Loss of *unc-38* in a *ced-8* background only very weakly restored PS exposure, if at all (79% in *ced-6* single mutant; 15,8% in *ced-6; ced-8* double mutant; 17,3% in *unc-38; ced-6; ced-8* triple mutant). We conclude that the effect of Ach signaling loss on PS exposure is largely, and possibly completely dependent on CED-8 function.

## Discussion

In this study we report our observation that loss of ACh signaling through the levamisole-receptor results in a reduction in the number of persistent cell corpses in worms suffering from a defect in apoptotic cell clearance. We show that the levamisole receptor likely acts cell autonomously in the dying germ cells, and propose that AChR activity modulates engulfment efficiency at least in part by modulating the kinetics or extent of PS exposure on the dying cells.

Even though *C*. *elegans* expression of *unc-38* and *unc-29* has only been reported in neuronal and muscular tissue, the fact that in vertebrates these channels can be expressed in further tissues, including epithelial cells and macrophages [26], brings up the possibility that this could also be the case in *C*. *elegans*. Unfortunately, we were unable to directly test this hypothesis since we failed, despite considerable effort, to obtain functional transgenes expressing UNC-38. To circumvent this limitation, we performed tissue-specific RNAi, which allowed us to determine that, surprisingly, expression of the levamisole receptor in germ cells was necessary and sufficient for its ability to modulate germ cell engulfment by the surrounding myoepithelial sheath cells. Taken at face value, these observations could suggest that apoptotic cells express nAChRs, and that modulation of ACh signaling can affect, directly or indirectly, the efficiency with which dying cells are engulfed by phagocytic cells. Mutations of other genes involved in ACh turn-over also reduce cell corpse numbers, supporting the hypothesis that ACh signaling is important for proper cell clearance. The source of the ACh that would be expected to act on the dying cells is at this point unclear.

It is known that dying cells expose phosphatidylserine on the outer leaflet of their plasma membrane as a sort of “eat-me” signal. In our study, we observed an increase in the fraction of dying cells exposing PS. This increase was dependent on the putative lipid scramblase CED-8. In mammals, PS exposure is known to be mediated at least partially by TMEM16F, a Ca^2+^ dependent phospholipid scramblase [46]. A recent report has further demonstrated that *anoh-1* a close homolog of TMEM16F in *C*. *elegans*, influences PS exposure in the surface of necrotic tail neurons [47]. It is attractive to speculate that ACh signaling modulates ionic concentrations at the plasma membrane of apoptotic cells and thereby may influence the dynamics of PS exposure by affecting the activity of one or several ion-sensitive scramblase proteins such as CED-8. This hypothesis might also explain why both loss of ACh signaling and excessive activation of the pathway lead to improved engulfment and increased PS exposure: both types of changes can be expected to interfere (in different ways) with normal ion fluxes and ion concentration changes at the plasma membrane, and thus might compromise the normal regulation of proteins such as CED-8.

Intracellular ion variation in engulfing cells has been shown to influence apoptotic cell clearance. In *Drosophila* calcium waves in engulfing cells have been reported to play a role in apoptotic cell uptake [21]. The work presented here opens the door to the possibility that corpse engulfment might also be influenced by ionic changes within the dying cell. Further studies on local ion concentration at the plasma membrane of the dying cell using genetically encoded ion reporter molecules in *C*. *elegans* might be key to decipher the molecular mechanisms behind our observations.

## Supporting Information

S1 Fig Loss of *unc-29* reduces cell corpse numbers in soma and germ lines of engulfment mutants. (A) Cell corpses were scored in the head region of freshly hatched L1 larvae of the indicated genotype. (B) Germ cell corpses were scored in adult animals of the indicated genotypes 12 hours post-L4/adult molt. *ced-x* stands for the genotypes on the x-axis. Data shown are average ± standard deviation, n = 20. All tests had a significance of *P*<0.005 with the exception of the case noted, *p<0.05, determined by *t*-test.
(TIF)

S2 Fig Apoptotic timing in *unc-29* mutants. The time of onset of the first 13 apoptotic cell deaths of the AB lineage were followed by 4D microscopy. Data shown are average ± standard deviation. Alleles: *unc-29(e1072)* and *ced-6(n1813)*.
(TIF)
