# Supplementary figures and images for: Loss of Acetylcholine Signaling Reduces Cell Clearance Deficiencies in Caenorhabditis elegans
Source: PLoS One. 2016 Feb 12;11(2):e0149274. doi: 10.1371/journal.pone.0149274 (PMC4752328; doi:10.1371/journal.pone.0149274)

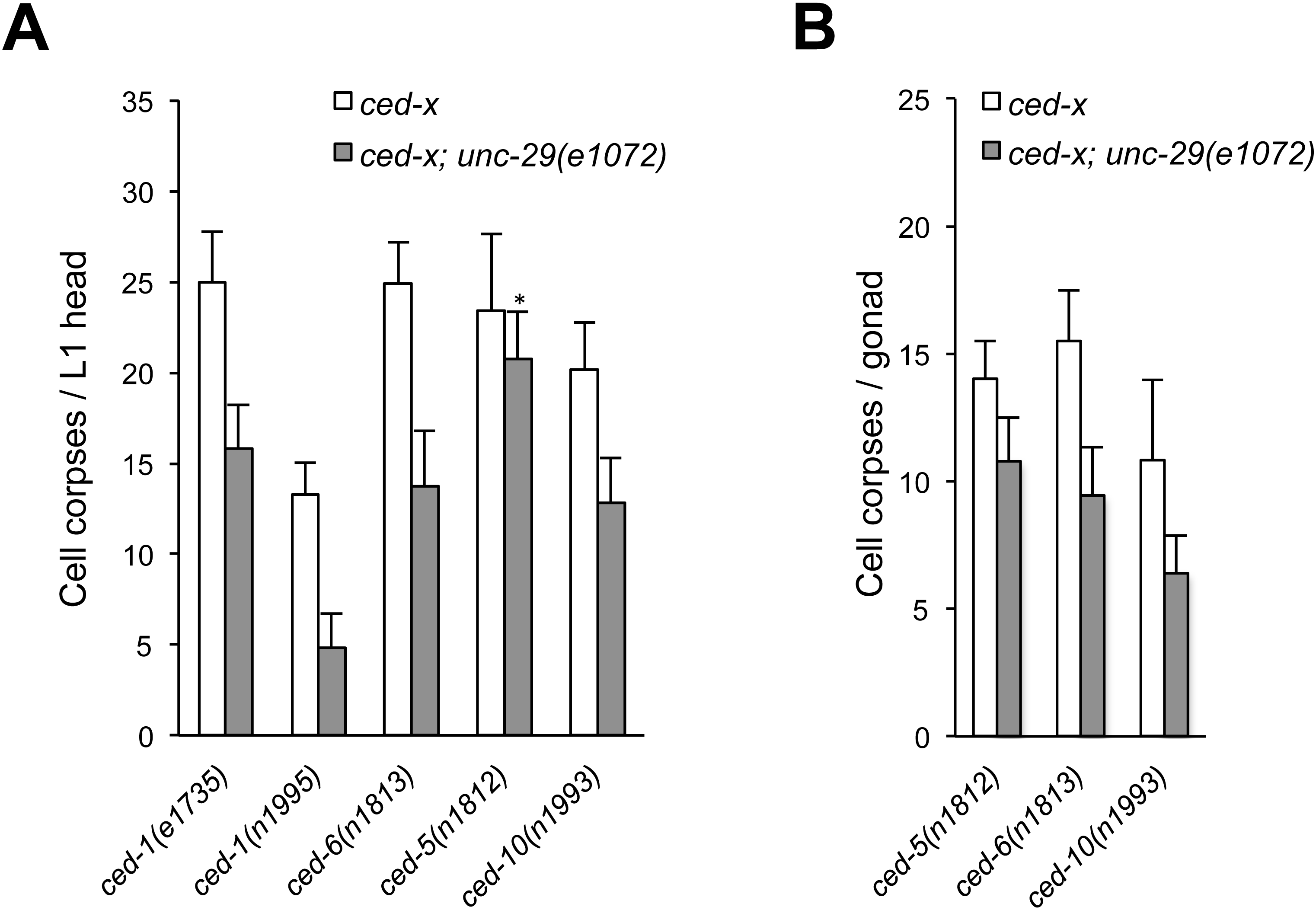

Supplement: S1 Fig — (A) Cell corpses were scored in the head region of freshly hatched L1 larvae of the indicated genotype. (B) Germ cell corpses were scored in adult animals of the indicated genotypes 12 hours post-L4/adult molt. ced-x stands for the genotypes on the x-axis. Data shown are average ± standard deviation, n = 20. All tests had a significance of P<0.005 with the exception of the case noted, *p<0.05, determined by t-test. (TIF) [file pone.0149274.s001.tif]

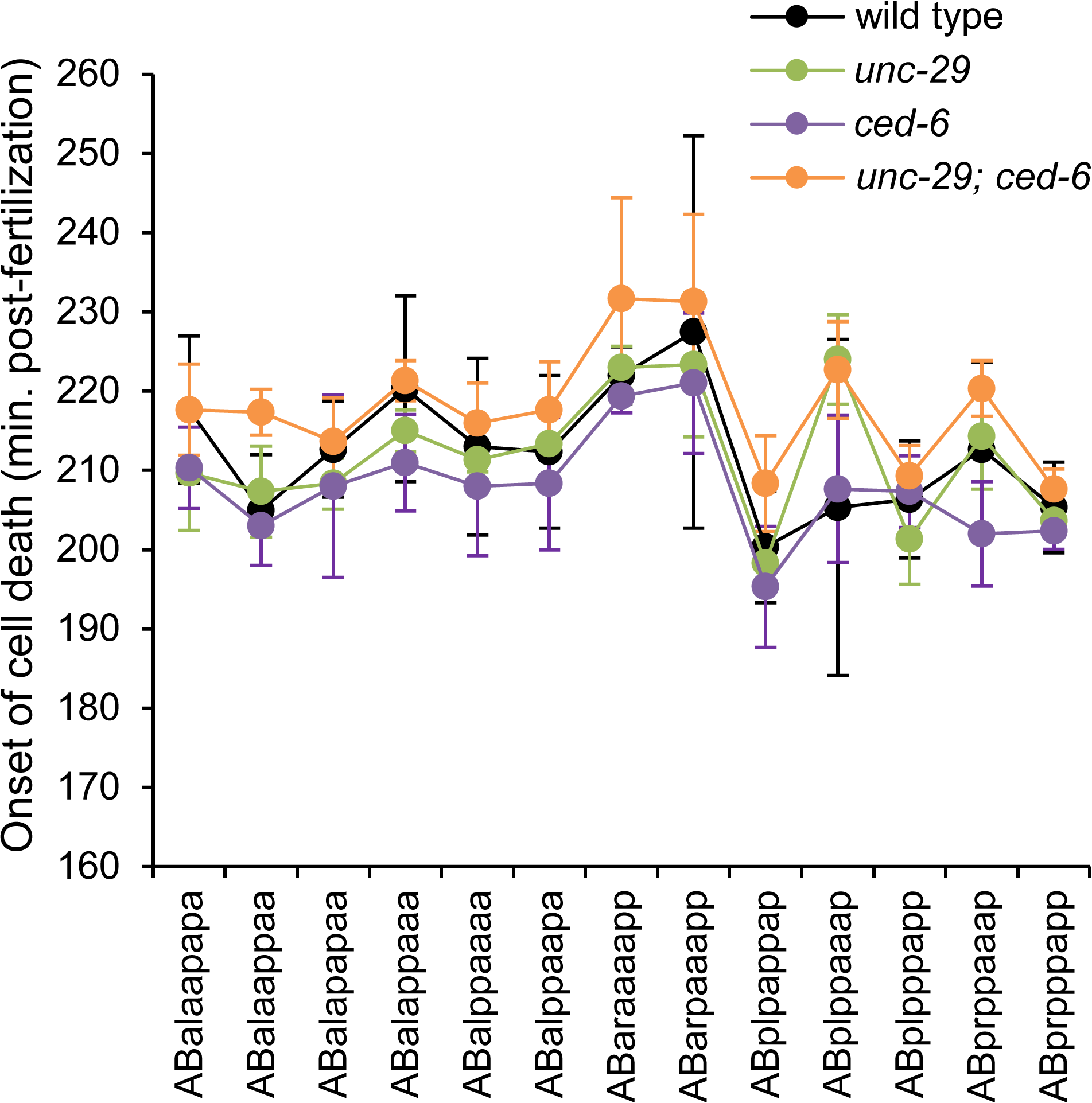

Supplement: S2 Fig — The time of onset of the first 13 apoptotic cell deaths of the AB lineage were followed by 4D microscopy. Data shown are average ± standard deviation. Alleles: unc-29(e1072) and ced-6(n1813). (TIF) [file pone.0149274.s002.tif]
